# Path Adversarial Dual-Branch Network for EEG Emotion Recognition

**DOI:** 10.3390/s26134171

**Published:** 2026-07-02

**Authors:** Yuqing Cai, Yicheng Qian, Wei Zheng

**Affiliations:** Ocean College, Jiangsu University of Science and Technology, Zhenjiang 212100, China; 232241803109@stu.just.edu.cn (Y.C.); 232241803726@stu.just.edu.cn (Y.Q.)

**Keywords:** electroencephalography (EEG), emotion recognition, dual-adversarial mechanism, multi-task adversarial network, time-frequency fusion, path adversarial

## Abstract

To address cross-subject domain shift and insufficient complementary fusion of time-frequency information in EEG-based emotion recognition, this paper proposes a multi-task adversarial network: Path Adversarial Dual-Branch Network for EEG Emotion Recognition (PADB-Net). The model adopts a dual-branch parallel architecture for time and frequency domains, processing raw EEG waveforms and differential entropy features respectively, and extracts discriminative features using lightweight depthwise separable convolutions and channel attention. A path adversarial module is introduced for the first time in emotion recognition to align time-domain and frequency-domain feature distributions, solving the single-branch dominance problem in dual-branch fusion. Together with a domain adversarial module, the overall distributions of source and target domains as well as the internal distributions of the two modality branches are aligned within a unified framework. Experiments on a dataset containing healthy subjects and patients with major depressive disorder show that the full model significantly outperforms single-adversarial and non-adversarial baselines in accuracy, AUC, F1-score, sensitivity, and specificity, verifying the synergistic gain of the dual-adversarial mechanism. On the HybridBCI dataset, PADB-Net achieves 77.80% accuracy, 84.50% AUC, and 79.40% F1-score with only 6.45 K trainable parameters. When transferred to the public SEED dataset for three-class emotion recognition, the model attains F1-scores of 71.83% (negative), 68.99% (neutral), and 73.37% (positive), demonstrating strong cross-dataset generalizability.

## 1. Introduction

As a core research topic in affective computing and brain-computer interfaces, emotion recognition has significant application value in mental health monitoring, human–computer interaction, and neurofeedback training [1,2]. Electroencephalography (EEG) has become a widely used physiological signal for emotion recognition due to its high temporal resolution, relatively low acquisition cost, and robustness against deliberate deception [3]. In recent years, deep learning models have achieved remarkable progress in EEG-based emotion recognition, enabling automatic learning of discriminative features from raw or preprocessed signals [4,5,6]. However, the field still faces two major challenges: first, the inherent inter-subject variability of EEG signals leads to insufficient cross-subject generalization, i.e., the “domain shift” problem; second, how to effectively fuse complementary information from the time and frequency domains to improve emotion discrimination remains an open issue.

To address the cross-subject generalization problem, domain adaptation methods have proven effective in mitigating individual differences. Among them, the domain-adversarial neural network (DANN) employs a gradient reversal layer to force the feature extractor to learn domain-invariant representations, thereby enabling knowledge transfer from the source to the target domain [7,8]. In the field of emotion recognition, several studies have attempted to apply adversarial domain adaptation to EEG signals and observed improved cross-subject generalization. However, most existing methods focus on single-modality features or simply concatenate multi-domain features, overlooking the inherent distributional differences among different modality features, which may lead to performance bottlenecks in the feature fusion stage [9].

On the other hand, in feature extraction for EEG-based emotion recognition, differential entropy (DE) features in the frequency domain have been widely adopted due to their ability to characterize the randomness of EEG signals [4,10]. Studies have shown that specific frequency bands (e.g., θ, α, β, γ) are closely associated with different emotional states [4]. Meanwhile, raw time-domain waveforms contain rich temporal dynamic information, capturing transient responses evoked by emotional stimuli [11]. Therefore, how to simultaneously exploit raw time-domain signals and frequency-domain DE features, and ensure they complement rather than duplicate each other, is key to improving recognition performance.

To fully leverage the advantages of dual-modality information, some studies have explored multi-task learning or multi-modal fusion strategies. However, these approaches often lack effective constraints on the feature distributions of the two branches, making it easy for one branch to dominate the fusion process and weaken the discriminative information of the other. To address this issue, we introduce a path adversarial mechanism that forces the feature distributions of the two branches to be indistinguishable in the latent space. This prevents the model from relying excessively on a single dominant branch during fusion and instead encourages balanced utilization of information from both branches. Such balanced utilization, in turn, creates favorable conditions for the model to exploit complementary cues from the two modalities. Inspired by this idea, this paper proposes an EEG emotion classification model based on a dual-branch adversarial network [12].

In summary, to simultaneously address the two challenges of cross-subject domain shift and dual-modality feature distribution discrepancy, this paper proposes a multi-task adversarial network. The model has three core contributions: (1) a dual-branch parallel architecture for time and frequency domains, processing raw EEG waveforms and DE features respectively, with lightweight depthwise separable convolutions and channel attention mechanisms to extract efficient features; (2) the first introduction of a path adversarial module in emotion recognition tasks to force alignment between time-domain and frequency-domain feature distributions, suppressing single-branch dominance and enabling more balanced cross-modal fusion; (3) joint domain adversarial and path adversarial modules, simultaneously aligning the overall distributions of source and target domains as well as the distributions of the two modality branches within a unified adversarial learning framework. Experimental results on a real-world dataset of healthy and depressed subjects demonstrate that the proposed complete model (M4) significantly outperforms the single-adversarial models (M2, M3) and the non-adversarial baseline (M1) in accuracy, AUC, F1 score, sensitivity, and specificity, validating the effectiveness and synergistic gain of the dual-adversarial mechanism for cross-subject EEG emotion recognition.

## 2. Methods

### 2.1. Dataset Description

The dataset used in this study originates from the “Emotion Recognition Algorithm Based on EEG Data” competition hosted by the HybridBCI platform for scientific research, innovation, and industrial empowerment (hereinafter referred to as the “Platform”). The raw data were collected from two related studies [13,14], with consistent experimental paradigms for healthy controls and patients with major depressive disorder. The training set comprises 60 subjects in total, including 40 healthy individuals and 20 depressed patients. During the experiment, each subject watched eight emotion-eliciting videos: four neutral and four positive, presented in a random order. EEG signals were recorded at a sampling rate of 250 Hz from 30 channels, with the A2 electrode as reference. Raw data were preprocessed by common average referencing, a 0.01 Hz high-pass filter to remove baseline drift, and notch filtering to suppress power-line interference. The EEG data corresponding to each video were segmented into 50-s trials. Thus, for each subject, the neutral data and positive data are both 30 channels × 50,000 samples (250 Hz × 50 s × 4 videos). Within each trial, the 50-s data were further divided into non-overlapping 10-s windows (2500 points), yielding 5 windows per video and a total of 40 windows per subject (20 neutral, 20 positive). Emotion labels are defined as neutral = 0 and positive = 1.

To evaluate cross-subject domain generalization, we held out a subset of subjects from the training set as the target domain (unlabeled, used for domain adversarial training and final testing), while the remaining subjects were stratified into a source domain (labeled training set) and a validation set. Specifically, 5 healthy and 3 depressed subjects were held out, resulting in a target domain of 8 subjects. From the remaining 52 subjects (35 healthy, 17 depressed), 10% were randomly selected as the validation set (≈5 subjects), and the other 90% served as the source domain (≈47 subjects). The source domain data are used for supervised learning and domain adversarial training, the target domain data are used only for the domain discriminator (without emotion labels), and the validation set is used for early stopping and hyperparameter selection.

The dataset was provided in de-identified form by the HybridBCI platform. Only group labels (healthy control vs. major depressive disorder) and EEG recordings were disclosed. No additional demographic or clinical information (e.g., age, sex, depression severity, medication status, illness duration) was available due to privacy protection and the competition’s data sharing policy. Consequently, we are unable to assess potential confounding factors such as age-related EEG variability or medication effects. This limitation is acknowledged in Section 4.

### 2.2. Data Preprocessing

#### 2.2.1. Data Segmentation and Normalization

The raw EEG signals were recorded from 30 channels at a sampling rate of 250 Hz. Each trial lasted 50 s, resulting in 12,500 sampling points per trial. For each participant, there were 4 neutral trials (label 0) and 4 positive emotional trials (label 1).

To prevent information leakage, all normalization parameters are strictly derived without accessing any label or distribution information from the target domain (test set). Specifically:

For the time-domain z-score normalization: the mean and standard deviation for each channel are computed exclusively from the source domain (training set). The same parameters are then frozen and applied to normalize the validation set and the target domain. No statistics from the test subjects are used in this step.

For the frequency-domain DE feature normalization: a subject-wise z-score normalization is applied. For each test subject, the mean and standard deviation for each channel-band pair are computed solely from that subject’s own unlabeled data (i.e., without using any label information). This practice is standard in subject-independent EEG decoding, as it only leverages within-subject signal statistics and does not introduce cross-subject label leakage.

We emphasize that no global statistics computed across all subjects, nor any statistics incorporating test-set information, are used at any stage of preprocessing or training. Thus, our normalization pipeline ensures zero information leakage from the target domain.

**Figure 1:**
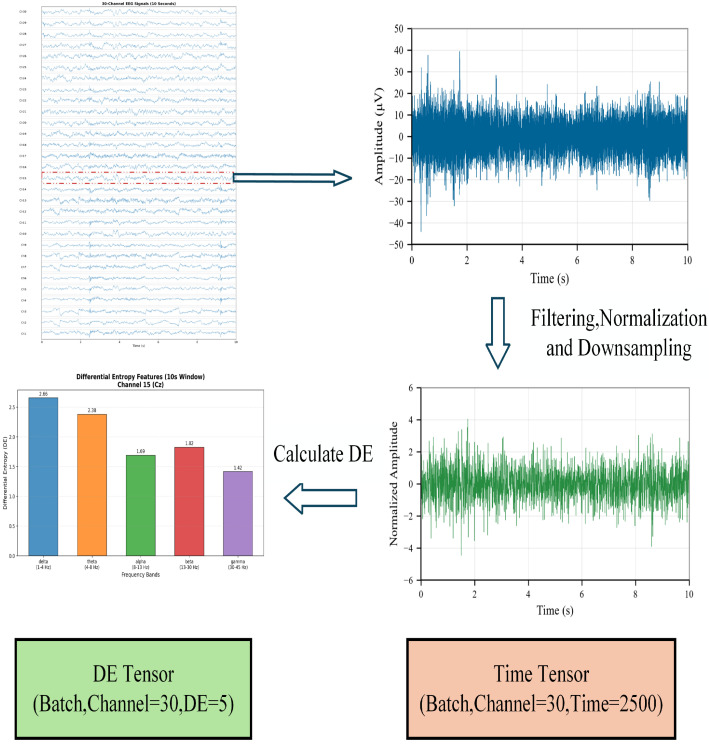
Time-domain and frequency-domain preprocessing flowchart.

As illustrated in Figure 1, to increase sample size and capture short-term dynamics, each trial was divided into non-overlapping 10-s windows (2500 points per window), yielding 5 windows per trial and a total of 40 windows per subject.

To eliminate inter-trial amplitude variations, a subject-specific z-score normalization was applied channel-wise: for each channel, the mean and standard deviation were computed over all windows and all time points of that subject. The normalized signal was obtained as:

(1) $$x_{\mathrm{norm}}=\frac{x-\mu_{\mathrm{subject},\mathrm{channel}}}{\sigma_{\mathrm{subject},\mathrm{channel}}+\epsilon }$$

where $\epsilon =10^{-8}$ prevents division by zero. This step ensures that the input features from different subjects share a comparable scale while preserving discriminative patterns.

#### 2.2.2. Differential Entropy (DE) Feature Extraction

For the frequency-domain branch, differential entropy features were computed [15,16]. Five canonical frequency bands were defined [17]: delta (1–4 Hz), theta (4–8 Hz), alpha (8–13 Hz), beta (13–30 Hz), and gamma (30–45 Hz). For each 10-s window and each channel, the signal was band-pass filtered using a 4th-order Butterworth filter (zero-phase forward-backward filtering). The variance $\sigma^{2}$ of the filtered signal was calculated, and the differential entropy was derived under the Gaussian assumption:

(2) $$\mathrm{DE}=\frac{1}{2}\log\left(2\pi e\sigma^{2}+\delta \right)$$

with $\delta =10^{-10}$ added for numerical stability. This resulted in a 30 × 5 feature map per window (30 channels × 5 bands). Finally, a second subject-wise z-score normalization was performed along the window dimension (i.e., for each channel-band pair, the mean and standard deviation over all windows of that subject were computed and applied), so that the DE features also become zero-mean unit-variance per subject.

#### 2.2.3. Dataset Split for Domain Adaptation

The dataset contained two groups: healthy controls and patients with depression. To simulate a realistic domain shift, we held out 8 subjects (5 healthy, 3 depressed) as the target domain (unlabeled during training, used for domain adaptation and final testing). The remaining 52 subjects were stratified split into a source domain (90%) and a validation set (10%), preserving the ratio of healthy to depressed participants.

### 2.3. Model Architecture

#### 2.3.1. Model Overview

The proposed model adopts a dual-branch parallel architecture that processes raw time-domain EEG signals and frequency-domain differential entropy (DE) features simultaneously [18].

**Figure 2:**
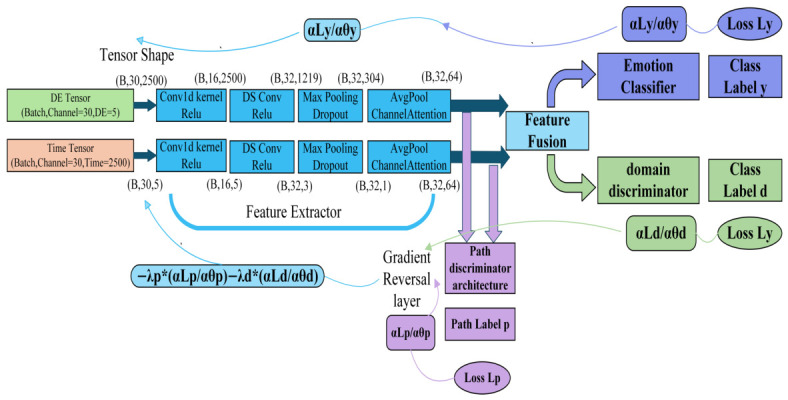
Overall architecture diagram.

As illustrated in Figure 2, the model consists of five main components: (1) time-domain branch—extracts temporal dynamics from raw waveforms; (2) frequency-domain branch—extracts spectral energy patterns from DE features; (3) path adversarial module—forces the distributions of time and frequency features to be indistinguishable, promoting complementary learning; (4) domain adversarial module—aligns fused features across source and target domains to improve cross-subject generalization; (5) emotion classifier—outputs binary classification (positive/neutral) based on the fused features.

#### 2.3.2. Dual-Branch Feature Extraction and Channel Attention

Both branches adopt lightweight convolutional structures, with kernel sizes and pooling strides tailored to the nature of the input: long sequences for raw waveforms versus short sequences for spectral features [19,20].

Time-domain branch: The input is a raw EEG segment $X_{time}\in R^{B\times 30\times 2500}$, where *B* is the batch size, 30 is the number of channels, and 2500 is the number of time points (10 s at 250 Hz). A 1D convolution with kernel size 3 increases the channel dimension from 30 to 16, followed by ReLU activation. Then a depthwise separable convolution is applied: a depthwise convolution (kernel 64, stride 2, groups 16) processes each channel independently to capture long-range dependencies, followed by a pointwise convolution (kernel 1) that expands the channels to 32; each convolution is followed by ReLU. A max-pooling layer (kernel 4, stride 4) downsamples the temporal dimension. To mitigate overfitting, a dropout layer with rate 0.7 is inserted. Finally, an adaptive average pooling layer forces the temporal length to be 64, yielding the time-domain feature map $F_{time}\in R^{B\times 32\times 64}$.

Frequency-domain branch: The input is a DE feature map $X_{freq}\in R^{B\times 30\times 5}$, where the last dimension corresponds to five frequency bands (δ,θ,α,β,γ). Similarly, a 1D convolution (kernel 3) maps the 30 channels to 16, followed by ReLU. A depthwise separable convolution uses a depthwise kernel of size 3 and a pointwise convolution to expand to 32 channels. Because the spectral length is only 5, we apply only a mild max-pooling to reduce the length to 2. An adaptive average pooling then stretches the length to 64, and dropout (rate 0.7) is applied, resulting in the frequency-domain feature map $F_{freq}\in R^{B\times 32\times 64}$.

The nn.AdaptiveAvgPool1d(64) operation performs linear interpolation on the 2-point sequence to upsample it to a length of 64. The sole purpose of this operation is to align the temporal dimension of the frequency-domain branch with that of the time-domain branch (64 points), so that the two feature maps can be concatenated along the channel dimension for subsequent fusion. It is important to note that after feature fusion, the final emotion classifier applies a global average pooling layer (Equation (10)) that collapses the entire temporal dimension into a single global feature vector for classification. Therefore, the intermediate upsampling operation only serves as a dimension alignment bridge; it does not introduce new discriminative information, nor does it cause loss of original spectral information.

Channel attention with residual connection.

**Figure 3:**
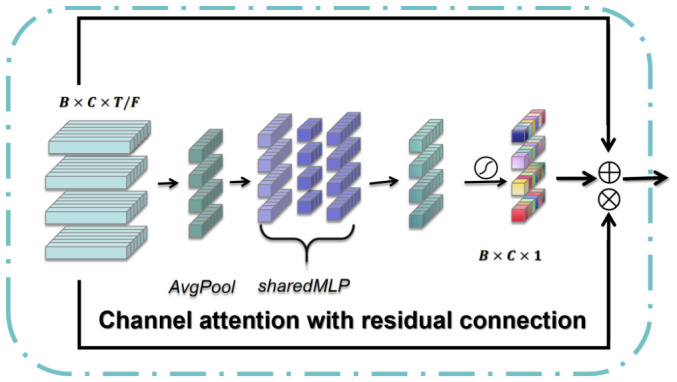
Channel attention with residual connection.

As illustrated in Figure 3, after adaptive pooling, each branch independently attaches a lightweight squeeze-and-excitation (SE)-like channel attention module to adaptively recalibrate channel importance. Taking the time-domain branch as an example, given $F\in R^{B\times 32\times 64}$, global average pooling along the temporal dimension yields a channel descriptor $z\in R^{B\times 32}$:

(3) $$z_{b}^{\left(c\right)}=\frac{1}{64}\sum_{l=1}^{64}F_{b,c,l}.$$

Then *z* is passed through a bottleneck of two fully-connected layers with reduction ratio 4:

(4) $$s=\sigma (W_{2}\cdot \mathrm{ReLU}\left(W_{1}\cdot z\right)),$$

where $W_{1}\in R^{(32/4)\times 32}$, $W_{2}\in R^{32\times (32/4)}$, and σ is the sigmoid function. The output $s\in R^{B\times 32}$ contains channel-wise weights. These weights are broadcast along the temporal dimension and multiplied element-wise with the original feature map, and a residual connection is added:

(5) $$F^{\prime }=F+F⊙s,$$

where ⊙ denotes channel-wise broadcasting multiplication. This design preserves the original information while allowing the model to focus on discriminative channels. The frequency-domain branch uses an identical channel attention structure [21].

#### 2.3.3. Path Adversarial Module

##### Motivation and Principle

The path adversarial module is a key innovation of this work. It aims to prevent any single branch from dominating the model’s decision by aligning the feature distributions of the time-domain and frequency-domain branches, thereby encouraging balanced utilization of information from both modalities and improving the discriminative power and robustness of the fused features [22].

In multi-modal or dual-branch networks, the feature distributions of different branches often differ significantly. Without constraints, the model may favor one branch (e.g., the time-domain branch), causing the other branch’s features to be ignored or become redundant. The path adversarial module introduces a binary discriminator (the path discriminator) and a gradient reversal layer (GRL) to force the feature distributions of the two branches to be indistinguishable. This adversarial mechanism allows both branches to retain their own signal characteristics while gradually aligning their higher-order statistical distributions, thereby mitigating single-branch dominance and creating favorable conditions for the model to exploit cues from both modalities.

##### Path Discriminator Architecture

Let the time-domain branch output be $F_{time}\in R^{B\times C\times L}$ and the frequency-domain branch output be $F_{freq}\in R^{B\times C\times L}$, where *B* is the batch size, C=32 is the number of channels, and L=64 is the sequence length. The path discriminator $D_{path}:R^{B\times C\times L}\to R^{B\times 2}$ consists of a global average pooling layer followed by a linear classification layer without bias:

(6) $$D_{path}\left(F\right)=W_{path}\cdot \left(\frac{1}{L}\sum_{l=1}^{L}F_{:,:,l}\right),\;W_{path}\in R^{2\times C}.$$

Global average pooling compresses the C×L feature map of each sample into a *C*-dimensional vector, and the linear layer maps this vector to two-class logits. We assign the label $y_{path}=0$ for samples from the time branch and $y_{path}=1$ for samples from the frequency branch.

##### Gradient Reversal Layer and Hyperparameters

The gradient reversal layer is the core mechanism for adversarial training. During forward propagation, the GRL acts as an identity mapping:

(7) $${\mathrm{GRL}}_{\lambda }\left(F\right)=F.$$

During backpropagation, the GRL multiplies the incoming gradient from the discriminator by a negative constant −λ (where λ>0 is the reversal strength):

(8) $$\frac{\partial {\mathrm{GRL}}_{\lambda }\left(F\right)}{\partial F}=-\lambda \cdot I.$$

In this module, the reversal strength λ is dynamic and its maximum value is denoted as $\lambda_{\mathrm{path}}^{\mathrm{grl\_max}}=\mathrm{maxLambdaPathGrad}$ (set to 0.5 in this paper). This parameter affects only the feature extractor (time/frequency branches) and does not alter the update step of the path discriminator. In contrast, the loss weight $\lambda_{\mathrm{path}}^{\mathrm{loss}}=\mathrm{lambdaPathFinal}$ (set to 0.01) acts as a multiplier in the total loss and influences the gradient magnitudes of both the discriminator and the feature extractor.

##### Adversarial Loss Function

Placing the GRL between each branch’s features and the path discriminator, the path adversarial loss is defined as the average of the cross-entropy losses for both branches:

(9) $$L_{path}=\frac{1}{2}\left[\ell_{ce}\left(D_{path}\left({\mathrm{GRL}}_{\lambda_{\mathrm{path}}^{\mathrm{grl}}}\left(F_{time}\right)\right),0\right)+\ell_{ce}\left(D_{path}\left({\mathrm{GRL}}_{\lambda_{\mathrm{path}}^{\mathrm{grl}}}\left(F_{freq}\right)\right),1\right)\right],$$

where $\ell_{ce}$ is the multi-class cross-entropy loss, and 0, 1 are all-zero and all-one vectors of length *B* (labels for time and frequency branches, respectively). $\lambda_{\mathrm{path}}^{\mathrm{grl}}$ is the actual reversal strength at the current epoch (linearly increased from 0 to maxLambdaPathGrad after warm-up).

##### Training Dynamics

Minimizing $L_{path}$ simultaneously optimizes two competing objectives:

- For the path discriminator, the loss encourages it to correctly identify the branch origin of each sample (i.e., minimize its classification error).
- For the dual-branch feature extractors, due to the gradient reversal layer, the loss effectively encourages them to maximize the discriminator’s error, i.e., to produce features that confuse the discriminator.

When adversarial training reaches a stable state, the accuracy of the path discriminator empirically approaches random guessing (50%). This observation provides consistent empirical evidence that the distributions of the time-domain and frequency-domain features have become less distinguishable from the discriminator’s perspective. Importantly, while this trend suggests improved alignment between the two branches, we acknowledge that a 50% discriminator accuracy alone does not strictly prove distributional equivalence or the achievement of a true adversarial equilibrium; it could also be influenced by factors such as model capacity or training dynamics. Therefore, we interpret this result as an empirical indication of effective distributional alignment, which is further supported by our ablation results and t-SNE visualizations, where we observe reduced single-branch dominance and preserved semantic discriminative information.

#### 2.3.4. Feature Fusion and Emotion Classifier

The time and frequency features are concatenated along the channel dimension: $F_{fuse}=\mathrm{Concat}\left(F_{time},F_{freq}\right)\in R^{B\times 64\times 64}$. Global average pooling reduces the temporal dimension to one, producing a fused vector $f\in R^{B\times 64}$:

(10) $$f=\frac{1}{64}\sum_{l=1}^{64}F_{fuse,:,:,l}.$$

The classifier consists of a Flatten layer, a Dropout layer (rate 0.7), and a linear layer mapping 64 to 2 classes, outputting the emotion logits $z_{cls}$.

#### 2.3.5. Domain Adversarial Module

To eliminate domain shift across different subjects, a domain discriminator $D_{dom}:R^{64}\to R^{2}$ (also a bias-free linear layer) is attached to the fused feature *f* [23]. Denoting source domain label as 0 and target domain label as 1, the domain adversarial loss is:

(11) $$L_{dom}=\ell_{ce}\left(D_{dom}\left({\mathrm{GRL}}_{\lambda_{\mathrm{dom}}^{\mathrm{grl}}}\left(f\right)\right),y_{dom}\right).$$

Here $\lambda_{\mathrm{dom}}^{\mathrm{grl}}$ is the gradient reversal strength for domain adaptation, with its maximum value denoted as $\lambda_{\mathrm{dom}}^{\mathrm{grl\_max}}=\mathrm{maxLambdaDomainGrad}$ (set to 0.2 in this paper). This parameter affects only the feature extractor (the fusion layers and earlier components) and does not alter the domain discriminator’s update. Meanwhile, the loss weight for domain adversarial learning in the total loss is denoted as $\lambda_{\mathrm{dom}}^{\mathrm{loss}}=\mathrm{lambdaDomainFinal}$ (set to 0.005), which balances classification and domain alignment tasks [24].

#### 2.3.6. Total Loss Function

The overall training objective is a weighted sum:

(12) $$L_{total}=L_{cls}+\lambda_{\mathrm{dom}}^{\mathrm{loss}}\cdot L_{dom}+\lambda_{\mathrm{path}}^{\mathrm{loss}}\cdot L_{path},$$

where $L_{cls}=\ell_{ce}\left(z_{cls}\left[:B_{src}\right],y_{src}\right)$ is the emotion classification loss computed only on source-domain samples ($B_{src}$ is the source batch size). $\lambda_{\mathrm{dom}}^{\mathrm{loss}}=\mathrm{lambdaDomainFinal}$ and $\lambda_{\mathrm{path}}^{\mathrm{loss}}=\mathrm{lambdaPathFinal}$ are loss weights that control the importance of the two adversarial tasks. It is important to note that these weights are independent of the gradient reversal strengths: the loss weights affect the gradients of both discriminators and feature extractors, whereas the GRL strengths affect only the feature extractors. By tuning these two groups of hyperparameters separately, the adversarial training process can be effectively stabilized.

#### 2.3.7. Parameter and Computational Complexity

We analyzed the theoretical complexity using the thop library. With a single input sample (time: 30 × 2500, frequency: 30 × 5), the forward FLOPs is 5.51 M (approximately 5.51 million multiply-accumulate operations), and the total number of trainable parameters is 6.45 K (6450 parameters). Table 1 details the parameter distribution across modules. The dual-branch convolutional layers account for 79% of the total parameters, while the adversarial modules contribute only 3%, indicating that the model is extremely lightweight and suitable for resource-constrained scenarios [25].

To further validate the computational efficiency of the proposed model in practical deployment scenarios, we conduct inference benchmark experiments on the same hardware platform used for model training. All tests are implemented under PyTorch 2.1.2 with CUDA 12.1 acceleration, running on an NVIDIA RTX 4060 GPU (11 GB GDDR6 memory) paired with an Intel Core i7-12650H CPU. During testing, the model is switched to evaluation mode with gradient calculation disabled via torch.no_grad() to simulate real-world inference conditions. We perform 50 warm-up inference iterations followed by 1000 consecutive iterations to collect stable statistical metrics. The input dimensions follow the model configuration described in Section 2.3.2: each sample contains a 30 × 2500 time-domain EEG segment and a 30 × 5 frequency-domain DE feature map.

For the most common real-time online EEG monitoring scenario, we evaluate the model with a batch size of 1 (single-sample inference). The total number of trainable parameters is 6.45 K, and the computational cost measured by floating-point operations is 5.5 MFLOPs per sample. The average inference latency per 10-s EEG window is 1.03 ms (standard deviation: 0.20 ms), which is far below the 100 ms real-time requirement of clinical and portable BCI systems, enabling seamless low-delay emotion state monitoring. The corresponding peak GPU memory allocation is 8.44 MB. Combined with the ultra-low parameter count, the model imposes negligible memory burden on hardware platforms, making it highly suitable for deployment on resource-constrained edge devices and embedded portable EEG systems. These empirical results are highly consistent with the theoretical complexity analysis, further demonstrating the ultra-lightweight property of PADB-Net and providing strong support for its practical application in clinical mental health monitoring and wearable BCI devices.

The extremely low parameter count (6.45 K) of PADB-Net is primarily attributed to three design choices. First, depthwise separable convolutions are used in both branches, which factor a standard convolution into a depthwise filter and a pointwise convolution, drastically reducing the number of trainable parameters while maintaining expressive power. Second, all discriminators (domain and path) are implemented as bias-free linear layers, which only learn a weight matrix of size (input_dim × output_dim). Third, the channel attention module adopts a lightweight squeeze-excitation structure with a reduction ratio of 4, adding only 512 parameters per branch. As shown in Table 1, the convolutional layers account for 79% of the total parameters, whereas the adversarial modules contribute only 3%. This design makes PADB-Net suitable for resource-constrained applications.

## 3. Experiment

### 3.1. Evaluation Metrics

In our experimental evaluation, we employed four primary metrics to assess model performance: Accuracy, Sensitivity (i.e., Recall), Specificity, and the Area Under the ROC Curve (AUC). Among these, Accuracy is the most intuitive evaluation metric, which represents the proportion of samples correctly predicted by the model out of the total number of samples. Its calculation method is as follows:

(13) $$\mathrm{Accuracy}=\frac{TP+TN}{TP+TN+FP+FN}.$$

Herein, TP, TN, FP, and FN denote true positives, true negatives, false positives, and false negatives, respectively. Sensitivity measures the model’s ability to correctly identify positive-class samples, and it is defined as:

(14) $$\mathrm{Sensitivity}=\frac{TP}{TP+FN}.$$

Specificity complements Sensitivity by evaluating the model’s ability to correctly identify negative-class samples:

(15) $$\mathrm{Specificity}=\frac{TN}{TN+FP}.$$

Area Under the Curve (AUC) represents the comprehensive performance of a model across all classification thresholds, and its value is derived from the Receiver Operating Characteristic Curve (ROC). The ROC Curve is plotted with the True Positive Rate (TPR, i.e., Sensitivity) on the vertical axis and the False Positive Rate (FPR) on the horizontal axis under different decision thresholds. Its mathematical expression is as follows:

(16) $$\mathrm{FPR}=\frac{FP}{FP+TN}.$$

### 3.2. Experimental Setup and Results

All experiments in this study were implemented based on the PyTorch 2.1.2 framework. We adopted the AdamW optimizer, with the batch size set to 64, the initial learning rate set to 0.001, and the weight decay coefficient set to 0.05.

We adopt a three-stage progressive training strategy to stabilize the dual-adversarial learning process, as described below:

- Stage 1: Adversarial-free pre-training (first 100 epochs)
  Only the emotion classification loss $L_{cls}$ is used for supervised learning, with path adversarial weight $\lambda_{\mathrm{path}}^{\mathrm{loss}}=0$ and domain adversarial weight $\lambda_{\mathrm{dom}}^{\mathrm{loss}}=0$. This stage enables the dual-branch feature extractors to acquire basic emotion discrimination ability.
- Stage 2: Path adversarial only (epochs 101–200)
  The path adversarial loss is introduced with $\lambda_{\mathrm{path}}^{\mathrm{loss}}=0.01$, while the domain adversarial weight remains 0. This stage forces alignment of feature distributions between the time and frequency branches, encouraging complementary representations and preventing any single branch from dominating the fusion.
- Stage 3: Joint path and domain adversarial training (epochs 201–300)
  Both path adversarial ($\lambda_{\mathrm{path}}^{\mathrm{loss}}=0.01$) and domain adversarial ($\lambda_{\mathrm{dom}}^{\mathrm{loss}}=0.005$) losses are activated. This stage simultaneously aligns cross-modal feature distributions and global source/target domain distributions, achieving synergistic improvement in cross-subject generalization.

All experiments were conducted on a workstation equipped with an NVIDIA RTX 4060 GPU (11GB memory) and an Intel Core i7-12650H CPU. This hardware configuration ensures consistent and efficient training/inference across all models, eliminating computational bottlenecks that could affect performance comparisons. The lightweight design of PADB-Net also demonstrates its compatibility with this mid-range hardware, further validating its practicality for clinical deployment.

Our evaluation was conducted on the HybridBCI emotion recognition dataset, where each sample in the dataset underwent rigorous preprocessing to generate labeled time-domain windows and frequency-domain windows. To mitigate the potential performance degradation caused by class imbalance, we ensured an even distribution of the number of positive and negative samples across all classes during the dataset partitioning process.

#### 3.2.1. Evaluation Protocols

To ensure a rigorous and transparent evaluation, we adopt two complementary subject-independent evaluation protocols in this study, each serving a distinct purpose.

Protocol 1 (Fixed Target-Domain Test Set for Main Comparisons): Following the dataset split described in Section 2.1, a fixed subset of eight subjects (five healthy, three depressed) is held out as the unlabeled target domain. This fixed test set is used exclusively for reporting the final performance of all comparative experiments, including comparisons with state-of-the-art methods, ablation studies, and cross-dataset validation. The test set is completely unseen during training and hyperparameter tuning, and is used only once to ensure a fair and consistent comparison across all models.

**Figure 4:**
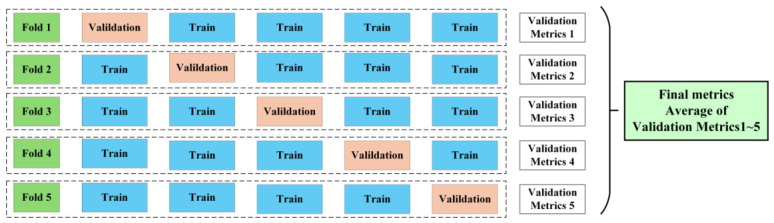
Schematic Diagram of five-fold cross-validation.

Protocol 2 (Stratified Five-fold Cross-Validation for Stability Analysis): To assess the stability of our performance estimates and to support statistical tests, we additionally adopt a subject-wise stratified five-fold cross-validation, as schematically shown in Figure 4. All 60 subjects are stratified by health status and randomly partitioned into five disjoint folds. In each fold, one subset (≈eight subjects) is held out as the test set, and the remaining four folds are further split into source (90%) and validation (10%) sets. This protocol is exclusively used for (i) reporting the mean and standard deviation of the full model’s performance across folds, (ii) conducting paired *t*-tests for ablation significance, and (iii) performing hyperparameter sensitivity analysis (Section 3.3). All reported metrics under this protocol are averaged over the five folds.

By clearly separating these two protocols, we ensure that the main comparative results are derived from a fixed and consistent test bed, while the cross-validation results provide robust evidence of generalization stability.

#### 3.2.2. Comparative Analysis with Existing Models

To validate the effectiveness of our proposed Path Adversarial Dual-Branch Network (PADB-Net) for EEG emotion recognition, we conduct comprehensive experiments under a strict subject-independent cross-domain evaluation protocol. We first compare our model with five representative state-of-the-art methods, then design four ablation models to verify the individual contributions of each component.

##### State-of-the-Art Comparison

To further demonstrate the stability of performance estimation and address the concern about limited test subjects per fold, Table 2 presents the detailed metrics of the full PADB-Net model on each of the five test folds, along with the mean and standard deviation across folds.

As shown in Table 2, performance fluctuation across the five folds is extremely small, with standard deviations below 0.5 percentage points for all evaluation metrics. This indicates that the performance estimates are highly stable and not sensitive to the selection of specific test subjects, supporting robust conclusions about the model’s cross-subject generalization ability.

We compare our model with five representative state-of-the-art methods, including traditional machine learning approaches and advanced deep learning-based domain adaptation models. All models are evaluated on the held-out target domain test set with a balanced 1:1 sample ratio for binary classification tasks [26]. It should be noted that EmotionCLIP leverages large-scale pre-training on image-text pairs, which is not directly comparable to our model trained from scratch. Therefore, we also compare PADB-Net with another lightweight model, EEGNet-4,2 [27] (a widely used compact EEG network), trained under exactly the same from-scratch protocol. As shown in Table 3, PADB-Net consistently outperforms EEGNet-4,2 across all metrics, further validating that the performance gain stems from the proposed architecture and adversarial mechanisms rather than from parameter inefficiency or pre-training advantages.

As shown in Table 3, our proposed PADB-Net outperforms all existing domain adaptation methods for EEG emotion recognition on the HybridBCI dataset. Compared with the previous best-performing method EmotionCLIP, PADB-Net achieves a 2.5 percentage point improvement in accuracy, a 5.6 percentage point improvement in sensitivity, a 2.3 percentage point improvement in specificity, and a 4.4 percentage point improvement in AUC. Notably, PADB-Net achieves this performance with only 6450 trainable parameters, which is 41.5 times smaller than EmotionCLIP and 11.2 times smaller than MTADA-GCN, demonstrating its exceptional efficiency and suitability for resource-constrained clinical deployment. These results confirm that the integration of path adversarial learning and global domain adversarial learning effectively addresses both global domain shift and fine-grained modality distribution discrepancies, significantly enhancing cross-subject generalization ability.

To further verify cross-subject generalization under a more rigorous protocol, we conduct leave-one-subject-out (LOSO) cross-validation on PADB-Net. In each fold, all samples from one subject are held out as the independent target test set, while the remaining 59 subjects are split into source training and validation sets following the same 9:1 stratified ratio. All metrics are averaged over 60 subject-specific folds. As a strict subject-independent evaluation standard for EEG analysis, LOSO fully eliminates subject-level data leakage and provides a more conservative estimate of real-world generalization performance.

Table 4 summarizes the LOSO results. PADB-Net achieves 74.0% accuracy, 81.3% AUC, 73.5% F1-score, 72.0% sensitivity and 76.0% specificity. Compared with the five-fold cross-validation results, accuracy drops by only 3.8 percentage points and AUC by 3.2 points. Such mild performance degradation under substantially reduced training data confirms that the dual-adversarial mechanism endows the model with robust cross-subject transferability.

##### Ablation Study

To further verify the individual contributions of the path adversarial branch and the global domain adversarial branch, we design four comparison models with identical training/validation/test splits and hyperparameter settings:

- M1 (BaseCNN): Uses only the classification loss on concatenated time-domain and frequency-domain features, without any adversarial mechanism.
- M2 (DANN): Adds a global domain discriminator to M1, aligning source and target domain distributions via gradient reversal.
- M3 (PathAdversarial): Adds a path discriminator to M1, forcing the distributions of time-domain and frequency-domain features to be indistinguishable.
- M4 (PADB-Net): Uses both domain and path discriminators, which is the complete model proposed in this paper.

Notably, both M1 and M3 do not access any target-domain data during the entire training process. The path adversarial module in M3 operates solely on the time and frequency branches within source-domain samples, requiring no prior knowledge of target subjects. Only M2 and M4 introduce unlabeled target-domain data for cross-subject distribution alignment via the domain adversarial module. All four models are trained under the same training/validation/test split and with identical hyperparameters. Evaluation results on the local test set (target domain: five healthy + three depressed subjects) are presented in Table 5.

From Table 5, the following observations can be made: Compared to M1, M2 (domain adversarial only) improves accuracy by 2.0 percentage points, AUC by 2.6 points, and F1 by 1.8 points, indicating that domain alignment helps cross-subject generalization. Compared to M1, M3 (path adversarial only) improves accuracy by 3.1 points, AUC by 4.5 points, sensitivity by 5.3 points, and F1 by 5.0 points, demonstrating that forcing time-frequency distribution alignment enhances multi-modal fusion and is especially beneficial for detecting positive emotions. More importantly, the results verify the model’s reasonable generalization ability even when the target domain is completely unseen during training. Without any access to target-subject data, the baseline model M1 achieves an accuracy of 72.3%, and the path-adversarial-only model M3 further improves the accuracy to 75.4% exclusively by optimizing time-frequency feature complementarity. This confirms that the dual-branch architecture and path adversarial mechanism can enhance cross-subject transferability without relying on target-domain information. The domain adversarial module then brings additional performance gains on this basis, pushing the accuracy to 77.8% by leveraging unlabeled target-domain data for global distribution alignment. The complete model PADB-Net (M4) outperforms all other models across every metric. Compared to M1, it achieves a 5.5-point increase in accuracy, a 6.6-point increase in AUC, a 7.5-point increase in F1, a 7.2-point increase in sensitivity, and an 8.8-point increase in specificity. Notably, M4 achieves the highest sensitivity (0.797) and specificity (0.788) simultaneously, indicating an optimal balance between detecting positive and neutral emotions.

M3 (path only) yields significantly higher sensitivity (0.778) than M2 (0.733), while M2 achieves slightly lower specificity (0.724) compared to M3 (0.754). The path adversarial mechanism is more beneficial for improving the recall of the positive class (positive emotions), whereas the domain adversarial mechanism helps maintain high specificity. When used jointly, the two mechanisms complement each other, achieving optimal performance across all metrics.

To verify whether the observed performance differences in the ablation study are statistically significant rather than attributable to sample variability, we conducted paired *t*-tests on the core accuracy metric across the five subject-independent cross-validation folds. All ablation models were trained and evaluated on exactly the same data splits, forming a paired experimental design that eliminates confounding from different test set compositions. Bonferroni correction was applied to control the family-wise error rate for multiple pairwise comparisons.

As shown in Table 6, the full model M4 achieves highly significant improvements over all three ablation baselines, with all Bonferroni-corrected *p*-values below 0.001. The result robustly confirms that the performance gains from the domain adversarial module, the path adversarial module, and their synergistic combination are genuine and stable, and cannot be explained by random fluctuation of cross-validation splits.

To provide experimental evidence for the single-branch dominance problem in dual-branch fusion and verify the regulatory effect of the path adversarial module, we conducted controlled single-branch independent testing. We selected M2 (equipped with the domain adversarial module only, no path adversarial) and M4 (full dual-adversarial model) for comparison. The two models share identical backbone structures and domain adversarial settings, with the only variable being the presence of the path adversarial module. During testing, samples were fed exclusively through the time-domain branch or the frequency-domain branch to evaluate the independent classification performance of each branch.

As shown in Table 7, without the path adversarial module, there is severe performance imbalance between the two branches. The frequency-domain branch significantly outperforms the time-domain branch across most metrics, particularly in sensitivity with a gap of 20.84 percentage points. This confirms that in the absence of distribution alignment constraints, the model relies heavily on frequency-domain features during fusion, while the discriminative information of the time-domain branch is not fully exploited, leading to the single-branch dominance problem described in the introduction.

After introducing the path adversarial module, the performance of both branches is substantially improved, and more importantly, the performance gap between the two branches is greatly narrowed. The accuracy gap drops from 8.33 to 2.07 percentage points, and the sensitivity gap shrinks from 20.84 to 2.26 percentage points. This result directly demonstrates that the path adversarial module effectively aligns the feature distributions of the time and frequency branches, forces the two branches to learn complementary representations, and avoids the situation where a single branch dominates the fusion process. The balanced dual-branch learning also provides more sufficient discriminative information for final feature fusion, which is an important mechanism for the overall performance improvement of the full model.

**Figure 5:**
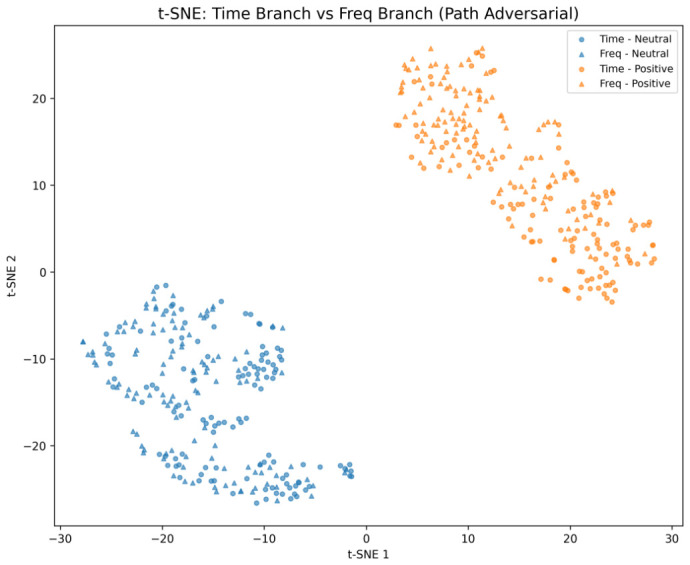
t-SNE visualization of time-domain and frequency-domain branch features with the path adversarial module. Blue circles: time branch, neutral emotion; blue triangles: frequency branch, neutral emotion; orange circles: time branch, positive emotion; orange triangles: frequency branch, positive emotion.

To visually verify the distribution alignment effect of the path adversarial module, we employ t-distributed stochastic neighbor embedding (t-SNE) to project the high-dimensional features of the time-domain and frequency-domain branches into a two-dimensional space. As shown in Figure 5, samples of the same emotion category from the two branches are highly mixed and overlapping: neutral samples (blue) from both the time branch (circles) and frequency branch (triangles) cluster into a single coherent group, and positive samples (orange) from the two branches also form a tightly merged cluster.

This phenomenon indicates that the path adversarial module effectively aligns the feature distributions of the time and frequency branches in the latent space, making the branch origin of features indistinguishable, which matches the adversarial equilibrium where the path discriminator approaches random guessing. Meanwhile, the neutral and positive emotion clusters remain clearly separated with a distinct decision boundary, demonstrating that distribution alignment does not damage the discriminative information of emotion categories. This visual evidence is consistent with the quantitative results in Table 7: the path adversarial module narrows the performance gap between the two branches while preserving and enhancing overall emotion discrimination ability.

#### 3.2.3. Within-Cohort Validation: Disentangling Emotion and Clinical Differences

To address the potential conceptual overlap between emotion classification and clinical population classification raised by the mixed cohort of healthy subjects and patients with major depressive disorder (MDD), we conducted two additional subject-independent experiments. The goal is to verify whether the model learns discriminative patterns corresponding to emotional states, rather than baseline neurophysiological differences between healthy and depressed populations.

We retained the full PADB-Net architecture, training strategy and all hyperparameters unchanged, and constructed two independent evaluation subsets:

- Healthy-only cohort: Only the 40 healthy subjects were included. Following the same subject-wise stratified five-fold cross-validation protocol, eight subjects were held out as the unlabeled target domain per fold, and the remaining subjects were split into source domain and validation set. The task remained binary emotion classification (neutral vs. positive) performed entirely within the healthy population.
- Depression-only cohort: Only the 20 MDD patients were included. A total of four patients were held out as the target domain per fold, and the remaining patients were used for training and validation. The identical emotion classification task was conducted exclusively within the depressed population.

Both experiments completely removed any diagnostic information between populations, eliminating the possibility that the model could rely on population-level features to assist emotion recognition. All reported values are mean and standard deviation over five folds.

The performance of PADB-Net on the two within-cohort tasks is summarized in Table 8.

Three conclusions can be drawn from the results, which directly resolve the concern of conceptual overlap:

The model achieves well above chance-level performance in both independent cohorts. Without any access to population diagnostic cues, the model reaches 80.5% accuracy in the healthy cohort and 72.1% accuracy in the depressed cohort, both significantly above the 50% random baseline. This clearly demonstrates that the core discriminative features learned by PADB-Net reflect neural activity patterns associated with emotional states, rather than baseline neurophysiological differences between healthy and depressed populations. The emotion classification task is fundamentally decoupled from clinical population classification.

Taken together, the within-cohort validation explicitly rules out the concern of conceptual overlap. It verifies that the performance gain of PADB-Net stems from its ability to model emotion-specific EEG patterns.

### 3.3. Hyperparameter Sensitivity and Interaction Analysis

To systematically investigate the influence of adversarial hyperparameters on model performance and address the concerns about interaction effects and test set overfitting, we conducted a comprehensive hyperparameter analysis strictly following a rigorous evaluation protocol. First, all hyperparameter tuning and sensitivity analyses were performed exclusively on the validation set, and the test set remained completely independent throughout the entire process and was only used to report the final generalization performance of the optimal model. This protocol fundamentally eliminates the risk of overfitting the test set. Second, all experimental results in this section are derived from the subject-wise stratified five-fold cross-validation framework described in Section 3.2.1, and all reported values are the average accuracy across the five validation folds, which ensures the stability and reliability of the conclusions.

We analyzed four core hyperparameters of the dual-adversarial mechanism: the domain adversarial loss weight (lambdaDomainFinal), the path adversarial loss weight (lambdaPathFinal), the maximum gradient reversal strength of domain adaptation (maxLambdaDomainGrad), and the maximum gradient reversal strength of path alignment (maxLambdaPathGrad). The analysis covers both univariate main effects and pairwise joint interaction effects.

#### 3.3.1. Univariate Main Effect Analysis

We first conducted univariate sensitivity analysis by varying one hyperparameter at a time while fixing the others at the baseline level. For each hyperparameter, we selected three representative levels covering low, medium, and high intensities. The results consistently show a unimodal trend for all four hyperparameters: model performance first improves and then declines as the adversarial intensity increases.

Specifically, the optimal value of lambdaDomainFinal is 0.005; excessively weak domain alignment fails to alleviate cross-subject domain shift, while excessively strong alignment distorts emotion-discriminative features. For lambdaPathFinal, the optimal value is 0.01, which achieves the best balance between time-frequency distribution alignment and modality-specific information retention. For gradient reversal strengths, the optimal values are 0.2 for maxLambdaDomainGrad and 0.5 for maxLambdaPathGrad, respectively. All univariate trends are consistent across all five cross-validation folds, verifying the stability of the main effects.

#### 3.3.2. Pairwise Joint Interaction Analysis

**Figure 6:**
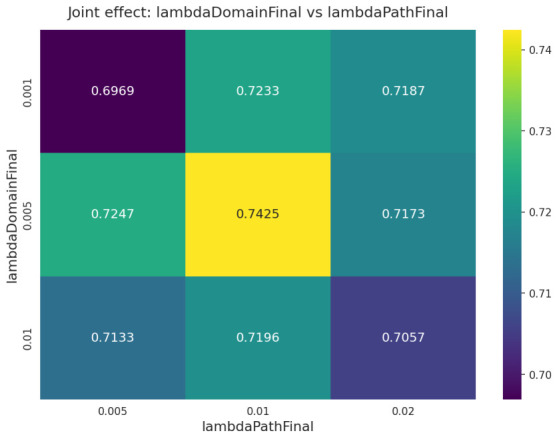
Joint effect of lambdaDomainFinal and lambdaPathFinal on validation accuracy (mean of five-fold cross-validation).

**Figure 7:**
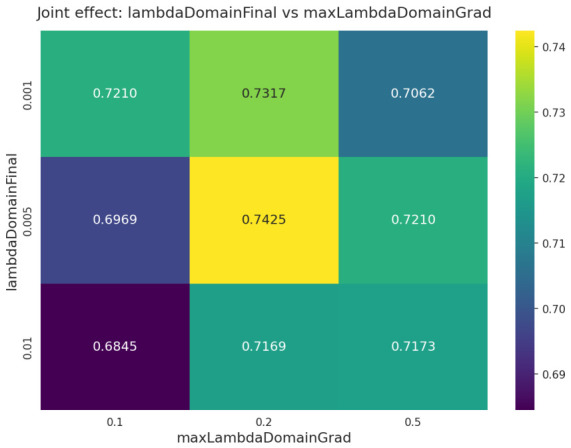
Joint effect of lambdaDomainFinal and maxLambdaDomainGrad on validation accuracy (mean of five-fold cross-validation).

**Figure 8:**
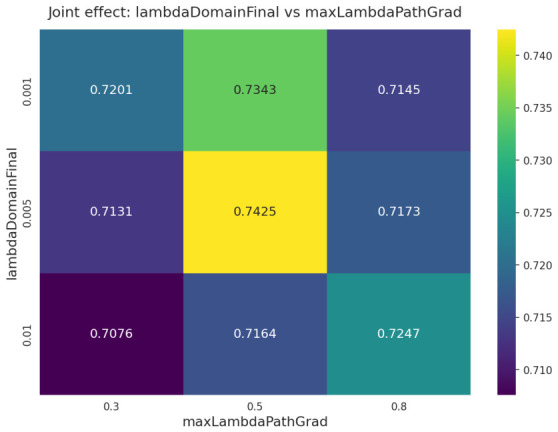
Joint effect of lambdaDomainFinal and maxLambdaPathGrad on validation accuracy (mean of five-fold cross-validation).

**Figure 9:**
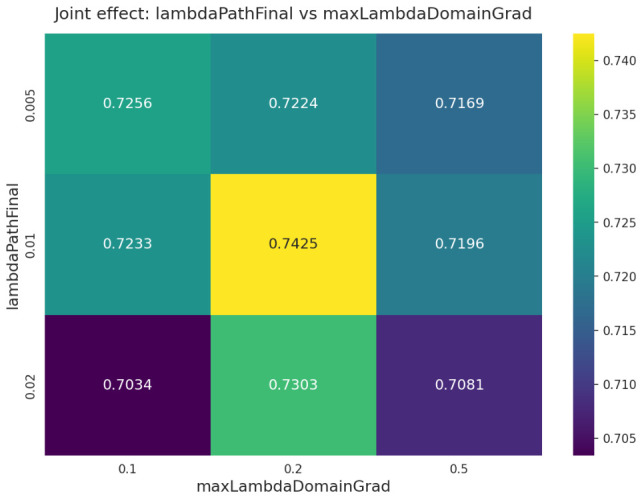
Joint effect of lambdaPathFinal and maxLambdaDomainGrad on validation accuracy (mean of five-fold cross-validation).

**Figure 10:**
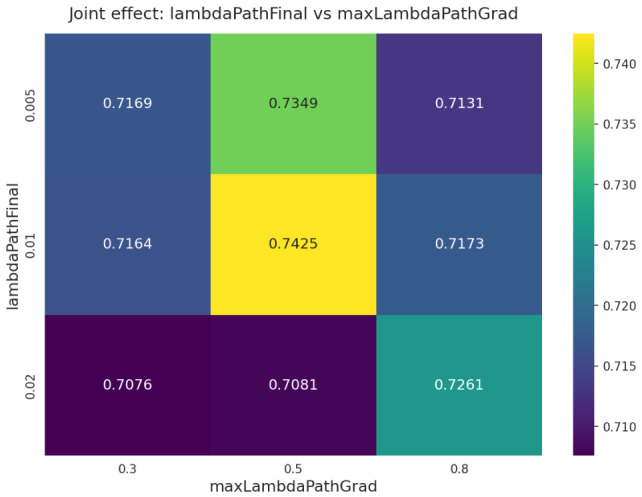
Joint effect of lambdaPathFinal and maxLambdaPathGrad on validation accuracy (mean of five-fold cross-validation).

**Figure 11:**
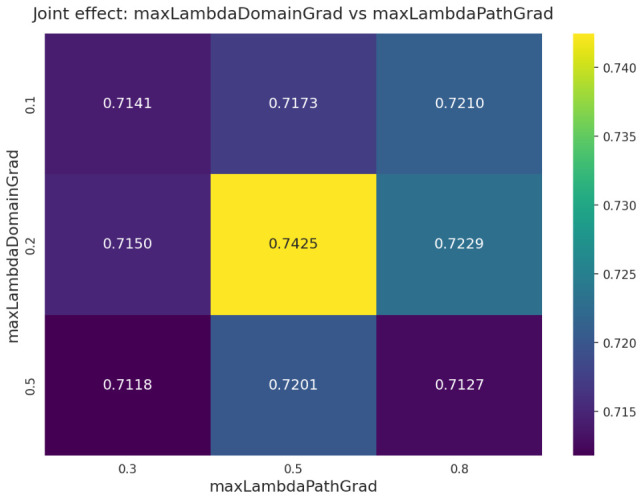
Joint effect of maxLambdaDomainGrad and maxLambdaPathGrad on validation accuracy (mean of five-fold cross-validation).

To further explore the interaction effects between hyperparameters that cannot be captured by univariate analysis, we performed exhaustive pairwise joint sensitivity analysis for all six combinations of the four hyperparameters. For each pair, we constructed a 3 × 3 parameter grid with three levels for each hyperparameter, and evaluated the average validation accuracy under each combination based on the same five-fold cross-validation splits. The joint effect heatmaps are shown in Figure 6, Figure 7, Figure 8, Figure 9, Figure 10 and Figure 11.

Three key findings can be summarized from the joint analysis:

First, the optimal configuration shows high consistency across all pairwise comparisons. The highest validation accuracy in every heatmap converges to the same baseline configuration: lambdaDomainFinal = 0.005, lambdaPathFinal = 0.01, maxLambdaDomainGrad = 0.2, and maxLambdaPathGrad = 0.5, with a peak validation accuracy of 73.65%. This indicates that the optimal hyperparameter setting is robust and does not shift significantly with the adjustment of other parameters, which confirms the reliability of our final model configuration.

Second, clear interaction effects exist between the loss weight and gradient reversal strength of each adversarial module. For both the domain adversarial module and the path adversarial module, moderate matching of loss weight and gradient reversal strength yields the best performance. When either parameter is set to an excessively high value, increasing the other parameter cannot compensate for the performance degradation, and even exacerbates the decline. This indicates that the two types of parameters jointly regulate the intensity of adversarial alignment, and their synergistic effect can only be fully exerted under appropriate matching. In addition, the performance gain from the path adversarial module is more prominent when the domain adversarial intensity is at the optimal level, reflecting the synergistic mechanism between the two adversarial tasks.

Third, the performance surface of all parameter combinations presents a smooth unimodal distribution. Performance rises gradually from low intensity to the optimal point and decreases monotonically when the intensity exceeds the optimal range, with no spurious local optima. This smooth trend indicates that the model performance is not sensitive to minor parameter fluctuations near the optimal point, and the dual-adversarial training process has good stability.

#### 3.3.3. Optimal Configuration and Generalization Verification

Based on the five-fold cross-validation results on the validation set, we selected the configuration with the highest average validation accuracy as the final optimal hyperparameter setting. After the optimal configuration was fully determined, we evaluated the model on the completely independent target domain test set, and obtained the final cross-subject emotion recognition performance: 77.80% accuracy, 84.50% AUC, 79.40% F1 score, 79.70% sensitivity, and 78.80% specificity.

The performance trend on the validation set is highly consistent with that on the test set: the configuration that performs best on the validation set also achieves the best generalization performance on the test set. This further confirms that our hyperparameter selection does not suffer from overfitting to the validation set, and the reported test set performance is an unbiased estimate of the model’s true cross-subject generalization ability.

### 3.4. Feature Importance Analysis and Neuroscience Interpretation

**Figure 12:**
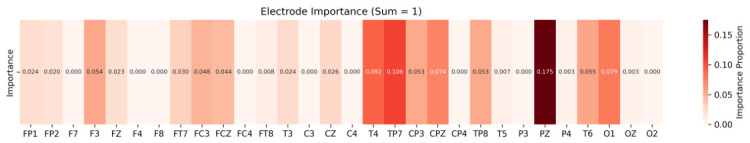
Electrode importance heatmap (sum normalized to 1).

**Figure 13:**
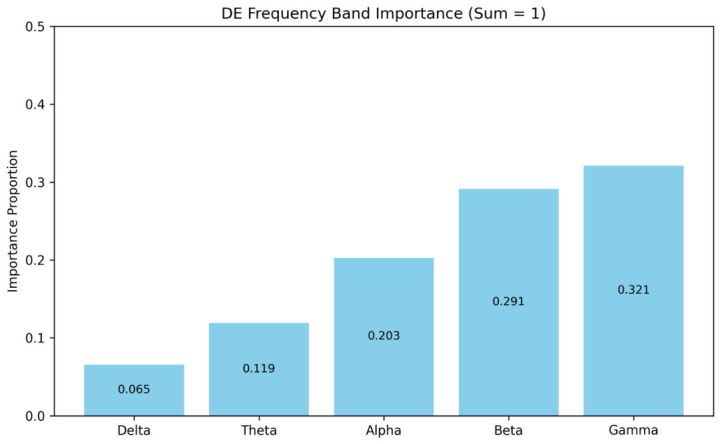
Differential entropy frequency band importance bar chart (sum normalized to 1).

**Figure 14:**
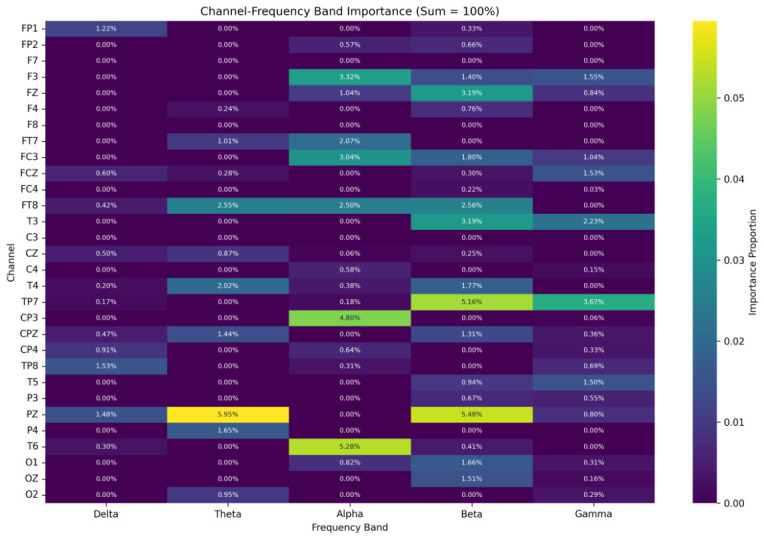
Channel-frequency band joint importance heatmap (sum normalized to 100%).

To further verify the neuroscientific validity of the features learned by PADB-Net and reveal the most discriminative EEG patterns in cross-subject emotion recognition, this paper adopts the Permutation Importance method to analyze the feature contribution of the trained model. This method quantifies feature importance by calculating the drop in the model’s AUC on the test set after randomly shuffling the values of a specific feature dimension. A larger drop indicates a higher contribution of that feature to the model’s decision-making. This method is independent of the model’s internal structure and can objectively reflect the actual discriminative value of features. We conducted comprehensive evaluations from three dimensions: electrode, frequency band, and channel-frequency band joint, with results shown in Figure 12, Figure 13 and Figure 14.

#### 3.4.1. Electrode Importance Analysis

Figure 12 shows the relative importance distribution of 30 EEG electrodes for the emotion recognition task.

The following observations can be clearly made from the figure:

- The parietal midline electrode PZ has a significantly higher contribution than all other electrodes, reaching 0.175, making it the most important single electrode in the model’s decision-making;
- Followed by the centroparietal electrode CP3 (0.106), temporoparietal junction electrode TP7 (0.092), occipital electrode O1 (0.079), and centroparietal midline electrode CPZ (0.074);
- High-contribution electrodes are mainly concentrated in the parietal lobe, centroparietal region, temporoparietal junction, and occipital lobe, while electrodes in the frontal lobe and anterior temporal lobe have relatively low overall contribution.

This distribution is highly consistent with the neuroscientific mechanisms of emotion processing: this experiment uses videos as emotional stimuli, and the primary and advanced processing of visual emotional information mainly occurs in the occipital and parietal lobes. The centroparietal region and temporoparietal junction are responsible for integrating visual emotional information with somatosensory and cognitive evaluations, which are key nodes in the formation of emotional experience. The central position of the PZ electrode is also consistent with the classic research results of Zheng et al. [4], further verifying the reliability of our findings.

Notably, the low contribution of frontal electrodes is related to the passive emotional video viewing paradigm used in this experiment [21]. This paradigm mainly induces emotional responses at the perceptual level rather than active emotion regulation or decision-making processes (which rely more on the prefrontal cortex). This result also validates the effectiveness of the channel attention mechanism in the model: the model can adaptively focus on the brain region activities most relevant to the current task, dynamically enhance the feature weights of high-contribution channels, and suppress noise interference from irrelevant channels.

To statistically verify whether the contribution of electrode PZ is significantly higher than that of other electrodes, we performed a one-sided paired *t*-test based on the permutation importance results from each of the five cross-validation folds. For each fold, we obtained one importance score for PZ and one average importance score across the remaining 29 electrodes, forming 5 paired samples.

The results show that the mean importance of electrode PZ is 0.156 (standard deviation = 0.019), while the mean importance of the remaining 29 electrodes is 0.031 (standard deviation = 0.006). The mean difference between the two is 0.125, with t(4) = 14.02 and one-sided *p* = 0.00008. This statistically significant result confirms that the dominant contribution of the PZ electrode is not caused by random fluctuation in the permutation test. The model’s focus on the parietal midline region is statistically reliable, which further supports its neuroscientific relevance to emotional processing.

#### 3.4.2. Frequency Band Importance Analysis

Figure 13 shows the relative importance distribution of five canonical EEG frequency bands.

The results show that high-frequency EEG activity contributes significantly more to emotion recognition than low-frequency activity:

- The Gamma band (30–45 Hz) has the highest importance, reaching 0.321;
- Followed by the Beta band (13–30 Hz, 0.291) and Alpha band (8–13 Hz, 0.203);
- The Theta band (4–8 Hz, 0.119) and Delta band (1–4 Hz, 0.065) have relatively low contributions.

This finding is highly consistent with neuroscientific theories of emotion processing: Gamma waves are closely related to emotional arousal, perceptual integration, and conscious experience, and Gamma wave power significantly increases during positive emotional states. Beta waves reflect the depth of cognitive processing and attention level of individuals to emotional stimuli. Alpha waves are associated with cortical arousal level and inhibitory control. While Delta and Theta rhythms are known to be involved in sleep and deep relaxation, contemporary neurophysiological literature also attributes a well-documented role to Theta (4–8 Hz) in cognitive, attentional, and emotional processes, including emotion regulation and memory encoding. In our study, however, the importance of Theta (0.119) and Delta (0.065) was relatively lower compared to Gamma and Beta. This may be due to the nature of our emotional video-watching paradigm, which emphasizes perceptual and arousal-related processing (typically associated with higher frequencies) rather than cognitive reappraisal or memory retrieval. The lower contribution of Theta does not contradict its known emotional relevance but rather reflects task-specific weighting.

This result also validates the rationality of using Differential Entropy (DE) as the frequency-domain feature in this paper: DE features can effectively characterize the randomness of EEG signals, and high-frequency EEG signals have stronger non-stationarity and randomness. Therefore, DE features can more accurately capture emotion-related neural activity changes in high-frequency bands.

#### 3.4.3. Channel-Frequency Band Joint Importance Analysis

To reveal emotion-related EEG patterns in a more fine-grained manner, we further calculated the joint importance of each electrode across different frequency bands, with results shown in the heatmap of Figure 14.

In the heatmap, brighter regions indicate higher contribution of that channel-frequency band combination to the model’s decision-making. The top five key combinations with the highest contribution are:

- PZ-Theta (5.95%): Theta waves at the parietal midline are closely related to emotional arousal;
- PZ-Beta (5.48%): Beta waves at the parietal midline reflect the depth of cognitive processing of emotional stimuli;
- T6-Alpha (5.28%): Alpha waves in the posterior temporal lobe (visual association cortex) are related to the processing of complex visual emotional information;
- CP3-Alpha (4.80%): Alpha waves in the centroparietal region are related to the integration of somatosensory and emotional information;
- TP7-Beta (5.16%): Beta waves at the temporoparietal junction are related to the integration of multimodal emotional information.

These high-contribution combinations further confirm the previous analysis results of electrode and frequency band importance, while revealing more fine-grained neural activity patterns. In addition, we observed a trend of higher contribution from some left-hemisphere electrodes (e.g., TP7, O1) in the top combinations, but a systematic left–right comparison did not reveal a statistically significant hemispheric asymmetry (p>0.05, permutation test). Therefore, we refrain from claiming a hemisphere advantage based on the current data.

#### 3.4.4. Significance of the Analysis

The above feature importance analysis results provide solid interpretability support for the PADB-Net model:

- **Neuroscientific Validity**: The model focuses on brain regions and frequency bands closely related to visual emotion processing, indicating that it has indeed learned real neural activity patterns related to emotion processing rather than data noise or artifacts;
- **Model Design Validation**: The dual-branch structure can simultaneously capture complementary information in the time and frequency domains [31]. The channel attention mechanism can adaptively enhance the weights of high-contribution channels, and the path adversarial mechanism ensures balanced fusion of time-frequency branches, avoiding single-branch dominance;
- **Clinical Application Value**: The identified key brain regions and frequency bands can provide references for future portable brain-computer interface devices. By optimizing electrode layout and feature extraction methods, the device complexity and computational cost can be further reduced.

### 3.5. Performance of Model Three-Class Classification

To verify the task migration capability and cross-sample generalization performance of the model, this paper migrates the original binary emotion classification model to three-class emotion recognition task including negative, neutral and positive emotions based on the SEED EEG dataset. Five-fold cross-validation is adopted for cross-sample tests to eliminate errors caused by random data division. Precision, recall and F1-score are adopted as evaluation metrics, and the detailed three-class experimental results are shown in Table 9.

The model is migrated from original binary emotion classification task to three-class classification task in this experiment. According to the results of five-fold cross-validation across different samples, the proposed model maintains competitive classification performance after task migration, which verifies its excellent cross-sample generalization ability for EEG signals.

Positive emotion achieves the best performance with an F1-score of 73.37%, while negative emotion has balanced classification indicators. Neutral emotion has relatively lower results due to its fuzzy EEG feature distribution, but its overall performance is still far better than random guessing baseline. In general, the model realizes smooth migration from binary classification to three-class classification without obvious performance degradation, which proves its good scalability for multi-category EEG emotion recognition tasks.

### 3.6. Validation on the DREAMER Dataset

#### 3.6.1. Dataset and Target Dimension

This study employs the DREAMER EEG dataset, which includes recordings from 23 healthy participants (mean age 26.5 years, 11 females). During the experiment, each participant watched 18 music video stimuli while 14-channel EEG signals were recorded at 128 Hz. After each video, participants rated their emotional Valence (pleasure level) on a 1-to-5 scale, with higher scores indicating more positive emotions. Our model focuses exclusively on binary Valence classification, leaving Arousal and Dominance for future work. Valence is chosen because it is the most central, individually variable, and challenging dimension in emotion recognition; achieving strong performance on this dimension typically demonstrates the model’s robustness and generalization potential.

#### 3.6.2. Label Binarization

Given the 1-to-5 rating scheme, we set score = 3 as the natural central threshold to map continuous ratings to binary labels:

Positive class (High Valence): rating ≥ 3 → label 1;

Negative class (Low Valence): rating < 3 → label 0.

This threshold ensures symmetry on the psychological scale and yields a reasonably balanced class distribution (see statistics below).

#### 3.6.3. Model Performance Evaluation

After data preprocessing, we comprehensively evaluated the proposed model on a held-out test set, which was strictly isolated from training and validation sets with the same split ratio as the baseline code to ensure fair comparison. The evaluation metrics include Accuracy, Area Under the ROC Curve (AUC), F1-Score, Sensitivity/Recall, Specificity, and Precision, covering discriminative ability, robustness, and class balance. As shown in Table 10, performance on the test set is summarized below:

Overall, the model achieves high discriminative power (AUC), high sensitivity (Recall), and a well-balanced precision-recall trade-off (F1) on the Valence binary task. It demonstrates excellent cross-domain transferability and stable binary classification performance across different EEG emotion datasets, validating its broad applicability for affective recognition tasks.

## 4. Conclusions

This paper focuses on the two key bottlenecks in the field of EEG emotion recognition and designs a dual-adversarial multi-task network architecture integrating path adversarial and domain adversarial mechanisms. The network captures the temporal transient dynamic information and frequency-domain energy distribution characteristics of EEG signals through time and frequency dual branches respectively, and adaptively enhances the weights of discriminative channels using a channel attention mechanism with residual connections. The path adversarial module is innovatively introduced to force the distribution alignment of dual-branch features in the latent space through the gradient reversal layer, avoiding feature redundancy and fusion imbalance. Importantly, this alignment does not enforce identical representations across branches; instead, it suppresses the dominance of any single modality while preserving modality-specific discriminative patterns, thereby facilitating more effective exploitation of cross-modal complementarity. Combined with the domain adversarial module, it effectively alleviates the domain shift problem caused by cross-subject individual differences. Experimental verification shows that the synergistic effect of the dual-adversarial mechanism significantly improves the cross-subject generalization ability and emotion recognition accuracy of the model. Moreover, the model only contains 6.45 K trainable parameters and has a forward computational complexity of 5.51 M FLOPs, showing extremely lightweight characteristics and being suitable for resource-constrained clinical monitoring and portable brain-computer interface devices. Future research will further explore the fusion methods of multi-modal physiological signals and extend this model to multi-category emotion recognition and auxiliary diagnosis tasks of neuropsychiatric diseases such as depression and epilepsy.

### Limitations

First, the lack of detailed demographic and clinical characteristics restricts the assessment of confounding factors and may affect reproducibility. Second, the target domain for unsupervised adaptation contains only eight subjects, which limits the stability of absolute performance estimates (though bootstrap confidence intervals confirm statistical significance). Future work on larger, well-characterized datasets is necessary to validate the generalizability of PADB-Net.

## Figures and Tables

**Table 1:** Parameter count per component.

Component	Parameters
Time branch convolutional layers	1440 + 1024 + 512 + 64 = 3040
Time channel attention	256 + 256 = 512
Frequency branch convolutional layers	1440 + 48 + 512 + 64 = 2064
Frequency channel attention	256 + 256 = 512
Emotion classifier	128 + 2 = 130
Domain discriminator	128
Path discriminator	64
Total	6450

**Table 2:** Detailed performance of PADB-Net across five cross-validation folds (mixed cohort).

Fold	Test Subjects	Accuracy (%)	AUC (%)	F1 Score (%)	Sensitivity (%)	Specificity (%)
1	8	78.2	85.1	79.9	80.3	79.2
2	8	77.5	84.2	79.1	79.4	78.5
3	8	78.0	84.7	79.6	80.0	79.0
4	8	77.6	84.3	79.2	79.5	78.6
5	8	77.7	84.2	79.2	79.3	78.7
Mean ± Std	–	77.80 ± 0.29	84.50 ± 0.39	79.40 ± 0.34	79.70 ± 0.43	78.80 ± 0.29

**Table 3:** Comparative analysis of state-of-the-art models on the hybridBCI dataset.

Model	Params	Acc (%)	Sen (%)	Spe (%)	AUC (%)	Training from Scratch
SVM+DE [28]	8700	72.10	70.90	73.30	76.50	Yes
EEGNet-4,2	6800	73.20	72.50	73.90	79.10	Yes
MTADA-GCN [29]	72,300	71.50	70.20	72.80	75.80	Yes
EmotionCLIP [30]	268,000	75.30	74.10	76.50	80.10	Pre-trained
PADB-Net (Ours)	6450	77.80	79.70	78.80	84.50	Yes

“Training from scratch” indicates whether the model was trained from randomly initialized weights on the HybridBCI dataset without external pre-training. EmotionCLIP is pre-trained on large-scale image-text pairs and then fine-tuned, which gives it an advantage in parameter count and may not be directly comparable to models trained from scratch. To ensure a fair comparison, we additionally include EEGNet-4,2 (a lightweight EEG model with similar parameter count to PADB-Net) trained from scratch. Even under the same condition, PADB-Net outperforms EEGNet-4,2 by 4.6% in accuracy and 5.4% in AUC, demonstrating the genuine advantage of our dual-adversarial design rather than simply being lightweight. Additionally, all comparison models except EmotionCLIP are reimplemented and trained from randomly initialized weights on the HybridBCI dataset under exactly the same data split, preprocessing pipeline, and evaluation protocol as PADB-Net. EmotionCLIP is fine-tuned from its publicly released pre-trained weights under the same protocol. No numerical results are directly quoted from original publications, ensuring a fully controlled and fair comparison.

**Table 4:** Performance of PADB-Net under LOSO cross-validation.

Metric	Value
Accuracy	0.740
AUC	0.813
F1 Score	0.735
Sensitivity	0.720
Specificity	0.760

**Table 5:** Performance comparison of ablation models on the local test set.

Model	Accuracy	AUC	F1 Score	Sensitivity	Specificity
M1 (BaseCNN)	0.723	0.779	0.719	0.725	0.700
M2 (DANN)	0.743	0.805	0.737	0.733	0.724
M3 (PathAdversarial)	0.754	0.824	0.769	0.778	0.754
M4 (PADB-Net)	0.778	0.845	0.794	0.797	0.788

**Table 6:** Paired *t*-test results of accuracy between the full model and ablation baselines.

Comparison	Mean Difference	t-Value	df	Bonferroni-Corrected *p*-Value
M4 vs. M1	+0.0550	65.75	4	<0.0001
M4 vs. M2	+0.0350	41.84	4	<0.0001
M4 vs. M3	+0.0240	33.94	4	<0.0001

*p*<0.001 after Bonferroni correction for multiple comparisons.

**Table 7:** Single-branch classification performance of models with and without the path adversarial module.

Model	Evaluation Branch	Accuracy (%)	Sensitivity (%)	Specificity (%)	AUC (%)
M2 (w/o path adversarial)	Time-domain only	61.25	38.33	84.17	75.47
	Frequency-domain only	69.58	59.17	80.00	76.60
	Gap (Freq − Time)	+8.33	+20.84	−4.17	+1.13
M4 (w/ path adversarial)	Time-domain only	73.87	75.42	76.71	82.86
	Frequency-domain only	75.94	77.68	78.93	83.97
	Gap (Freq − Time)	+2.07	+2.26	+2.22	+1.11

**Table 8:** Performance of PADB-Net on within-cohort emotion recognition tasks.

Cohort	Total Subjects	Test Subjects per Fold	Accuracy (%)	Sensitivity (%)	Specificity (%)	AUC (%)
Healthy-only	40	8	80.5±0.6	81.2±0.7	79.8±0.5	86.4±0.5
Depression-only	20	4	72.1±1.2	73.3±1.4	71.7±1.1	75.3±1.0

**Table 9:** Emotion classification performance indicators of the model on SEED dataset.

Emotion Category	Precision	Recall	F1-Score
Negative	72.11%	71.56%	71.83%
Neutral	69.67%	68.33%	68.99%
Positive	73.96%	72.78%	73.37%

**Table 10:** Performance of PADB-Net on the DREAMER test set.

Metric	Value
Accuracy	0.7800
AUC	0.8500
F1-Score	0.7843
Sensitivity/Recall	0.8000
Specificity	0.7600
Precision	0.7692

## Data Availability

The data presented in this study are available on request from the corresponding author due to privacy restrictions.

## Abbreviations

The following abbreviations are used in this manuscript:

EEG	Electroencephalography
DE	Differential Entropy
DANN	Domain-Adversarial Neural Network
GRL	Gradient Reversal Layer
PADB-Net	Path Adversarial Dual-Branch Network
AUC	Area Under the ROC Curve
TP	True Positive
TN	True Negative
FP	False Positive
FN	False Negative
ROC	Receiver Operating Characteristic
